# Phylogenetic analysis of symbionts in feather-feeding lice of the genus *Columbicola*: evidence for repeated symbiont replacements

**DOI:** 10.1186/1471-2148-13-109

**Published:** 2013-05-31

**Authors:** Wendy A Smith, Kelly F Oakeson, Kevin P Johnson, David L Reed, Tamar Carter, Kari L Smith, Ryuichi Koga, Takema Fukatsu, Dale H Clayton, Colin Dale

**Affiliations:** 1 Department of Biology, University of Utah, 257 South 1400 East, Salt Lake City, UT 84112, USA; 2 Illinois Natural History Survey, University of Illinois, 1816 S. Oak Street, Champaign, IL 61820, USA; 3 Florida Museum of Natural History, University of Florida, Gainesville, FL 32611, USA; 4 Bioproduction Research Institute, National Institute of Advanced Industrial Science and Technology (AIST), Tsukuba 305-8566, Japan

**Keywords:** Symbiosis, Insect, Lice, Co-speciation, Symbiont replacement

## Abstract

**Background:**

Many groups of insects have obligate bacterial symbionts that are vertically transmitted. Such associations are typically characterized by the presence of a monophyletic group of bacteria living in a well-defined host clade. In addition the phylogeny of the symbiotic bacteria is typically congruent with that of the host, signifying co-speciation. Here we show that bacteria living in a single genus of feather lice, *Columbicola* (Insecta: Phthiraptera), present an exception to this typical pattern.

**Results:**

The phylogeny of *Columbicola* spp. symbionts revealed the presence of three candidate clades, with the most species-rich clade having a comb-like topology with very short internodes and long terminal branches. Evolutionary simulations indicate that this topology is characteristic of a process of repeated symbiont replacement over a brief time period. The two remaining candidate clades in our study exhibit high levels of nucleotide substitution, suggesting accelerated molecular evolution due to relaxed purifying selection or smaller effective population size, which is typical of many vertically transmitted insect symbionts. Representatives of the fast-evolving and slow-evolving symbiont lineages exhibit the same localization, migration, and transmission patterns in their hosts, implying direct replacement.

**Conclusions:**

Our findings suggest that repeated, independent symbiont replacements have taken place over the course of the relatively recent radiation of *Columbicola* spp. These results are compatible with the notion that lice and other insects have the capability to acquire novel symbionts through the domestication of progenitor strains residing in their local environment.

## Background

Many insects maintain obligate, primary endosymbiotic bacteria that provide nutrients that are lacking in their natural diet. Associations between primary symbionts and their insect hosts are often ancient in origin, and have facilitated the exploitation of new ecological niches by insects [1]. The vertical transmission of primary symbionts often results in host-symbiont co-speciation, as evidenced by topological congruence between the insect and bacterial phylogenies [2,3].

Feather lice (Insecta: Phthiraptera) are obligate, permanent ectoparasites of birds and mammals that spend their entire life cycle on the host [4]. The genus *Columbicola* contains 88 described morpho-species, all of which parasitize columbiform birds (pigeons and doves) [5]. Some of these morpho-species are further divided into molecularly distinct cryptic species [6]. Species of *Columbicola* are relatively host-specific, with most known from only a single species of bird host. Transmission of lice between birds occurs mainly during periods of direct contact, as occurs between parent birds and their offspring in the nest [7]. However, *Columbicola* are also known to disperse phoretically on hippoboscid louse flies, which are winged parasites of birds [7,8]. True to their name, feather lice feed primarily on feathers, secretions, dead skin and other dermal “debris” [9]. Feathers present a nutritionally challenging diet because they consist mostly of keratins, which are difficult to digest and have amino acid compositions that are markedly biased [10]. In addition, the availability of vitamins and co-factors is expected to be limited in a diet comprising mostly feathers [11].

While a bacterial endocellular symbiont was observed microscopically in the abdomen of *Columbicola columbae* in 1931 [12], sequencing and phylogenetic analysis only recently revealed that this bacterium is a close relative of the tsetse fly symbiont, *Sodalis glossinidius*, which is in turn is a member of a well-established symbiont clade that is found in a diverse range of insect hosts [13]. *In situ* hybridization experiments demonstrated that the symbiont of *C. columbae* is housed within specialized bacteriocytes and passed to offspring via maternal (ovarial) transmission [13]. The function of the *C. columbae* symbiosis is currently undefined, but it seems likely that the symbiosis has a nutritional basis because of the fact that keratin-rich feathers represent a nutritionally incomplete diet [14].

The purpose of the current study was to perform a broad characterization of bacterial symbiont diversity in more than 40 members of the genus *Columbicola*, obtained from pigeons and doves collected in a worldwide survey*.* Since *Columbicola* symbionts are endocellular in bacteriocytes, we tested whether these symbionts exhibit patterns of co-speciation typical of long established, obligate associations found in other insects. However, in contrast, our molecular phylogenetic analyses revealed striking diversity and evolutionary dynamics in the host-symbiont associations of this single insect genus. We propose and test several hypotheses to account for these unexpected findings.

## Results

### Identification of *Columbicola* spp. symbionts

We initially sequenced 48 16S rRNA clones from individuals of *C. columbae* and *C. baculoides*. Only a single 16S rRNA sequence was identified from each host species. For each of the other *Columbicola* spp. in the study, we sequenced a minimum of four 16S rRNA gene clones. No within species sequence heterogeneity was observed, indicating that each of the *Columbicola* spp. screened in this study harbors only a single bacterial symbiont.

### Structural analysis of 16S rRNA sequences of *Columbicola* spp. symbionts

In the initial 16S rRNA gene phylogeny containing all of the sequences derived from the symbionts of *Columbicola* spp., the sequences derived from *C. veigasimoni* and *C. paradoxus* exhibited unusually long branches, indicating substantially higher evolutionary rates than the sequences of the other *Columbicola* spp. symbionts of the same clade (Additional file 1). In addition, the *C. veigasimoni* and *C. paradoxus* symbiont 16S rRNA sequences exhibited unusually low G + C contents relative to the other members of the same clade (Additional file 1). These patterns suggest the possibility that these highly divergent sequences might represent non-functional copies of the 16S rRNA gene in these symbiont genomes.

Secondary structure analyses of the 16S rRNA sequences using a homology model [15] revealed that the *C. veigasimoni* and *C. paradoxus* symbiont 16S rRNA sequences exhibit unusually high ratios of disruptive:conservative nucleotide substitutions. For example, in the *C. veigasimoni* 16S rRNA sequence, 85 out of a total of 180 substitutions (47.2%) are predicted to encode disruptive changes (causing putative stem-loop transitions; Additional file 2). Similarly, in the *C. paradoxus* 16S rRNA sequence, disruptive substitutions comprise 30 out of a total of 98 substitutions (30.6%; Additional file 3). In contrast, the *C. columbae* 16S rRNA sequence that resides on a relatively short branch has only 9 out of a total 69 substitutions (13%) that are characterized as disruptive (Additional file 4). Indeed, statistical analyses show that the ratios of disruptive/conservative substitutions in both the *C. veigasimoni* and *C. paradoxus* symbiont 16S rRNA sequences are significantly higher than in the *C. columbae* symbiont 16S rRNA sequence (Fisher’s exact test; *P* < 0.001). Furthermore, using the 16S rRNA variability map derived by Wuyts *et al*. [16], we determined that the *C. veigasimoni* and *C. paradoxus* symbiont 16S rRNA sequences also have significantly more substitutions in sites that normally display low variability (Fisher’s exact test; *P* < 0.0001). All these data suggest that the *C. veigasimoni* and *C. paradoxus* symbiont 16S rRNA sequences are not evolving in accordance with the functional constraints that affect other 16S rRNA sequences, including that of the *C. columbae* symbiont. However, it should also be noted that these sequences are remarkably free of indels, which have recently been shown to accumulate rapidly in the pseudogenes of *Sodalis*-allied symbionts [17]. Thus, based on the available data, we cannot determine if the highly disrupted 16S rRNA obtained from the *C. veigasimoni* and *C. paradoxus* symbionts are functional. Because of the possibility that the *C. veigasimoni* and *C. paradoxus* symbionts maintain additional (functional) paralogous copies of 16S rRNA that were not amplified by the universal primers used in this study, we elected to exclude the *C. veigasimoni* and *C. paradoxus* symbiont 16S rRNA gene sequences from subsequent molecular phylogenetic analyses.

### Phylogenetic analysis of 16S rRNA gene sequences of *Columbicola* spp. symbionts

**Figure 1:**
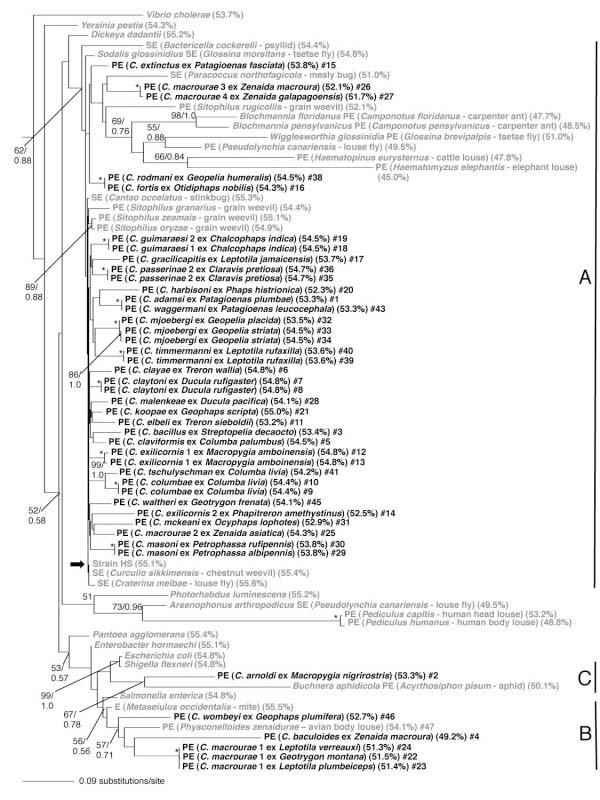
**16S rRNA phylogeny of *Columbicola *spp. symbionts.** Phylogeny of *Columbicola* spp. symbionts (bold) and related bacteria based on maximum likelihood and Bayesian analyses of a 1.46-kbp fragment of 16S rRNA gene sequences. Insect symbionts are designed by the prefix “PE” (primary endosymbiont), “SE” (secondary endosymbiont) or “E” (if unknown), followed by insect host name and common name (or latin name of bird host for *Columbicola* spp.) The numbers adjacent to nodes indicate maximum likelihood bootstrap values (to left of diagonal line) and Bayesian posterior probabilities, where applicable (to right of line), for nodes with bootstrap support >50% and Bayesian posterior probabilities >0.5. Asterisks indicate nodes with 100% bootstrap support and Bayesian posterior probability = 1. The bold arrow highlights the location of the sequence derived from strain HS, the recently characterized progenitor of the *Sodalis*-allied symbionts. Numbers in parentheses represent the G + C content of the 16S rRNA sequences. Final numbers correspond to sample numbers in Additional file 5.

In the 16S rRNA phylogeny, the symbionts of *Columbicola* spp. were assigned to three clades in the Gammaproteobacteria (Figure 1). At this stage, because of the relative paucity of representation in clades B and C, it should be noted that these designations are tentative, and not supported by extremely high bootstrap values. Clade A contains the largest number of *Columbicola* spp. symbionts and is represented by the symbiont of *C. columbae*, the tsetse fly symbiont (*Sodalis glossinidius*), and symbionts of grain weevils of the genus *Sitophilus*. Clade A also contains several long established primary endosymbionts including *Wigglesworthia glossinidia* and *Blochmannia* spp., further supporting the notion of a monophyletic origin of these bacteria and the *Sodalis*-allied symbionts [18]. Clade B is represented by several *Columbicola* spp. symbionts, including the symbiont of *C. baculoides*, and symbionts identified from the avian body louse *Physconelloides zenaidurae* and the mite *Metaseiulus occidentalis*[19]. The sole representative of clade C, the symbiont of *C. arnoldi*, is distantly allied to the aphid primary endosymbiont *Buchnera aphidicola*.

### Phylogenetic analysis of multiple gene sequences of *Columbicola* spp. symbionts

**Figure 2:**
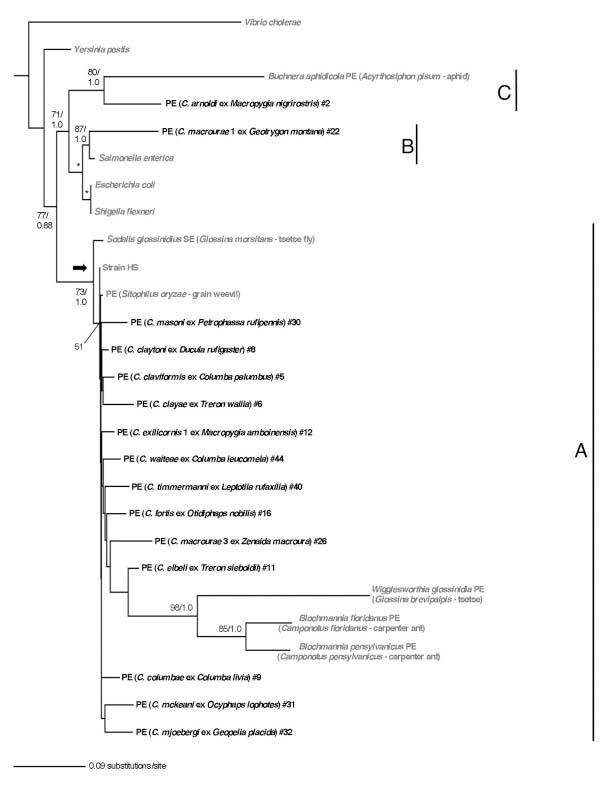
**Multigene phylogeny of *Columbicola *spp. symbionts.** Phylogeny of *Columbicola* spp. symbionts derived from maximum likelihood and Bayesian analyses of a combined data set consisting of 16S rRNA, *fusA* and *groEL* gene sequences. Conventions as in Figure 1.

On the basis of combined sequence data of 16S rRNA, *fusA* and *groEL* genes, the symbionts of *Columbicola* spp. were also divided into three distinct clades A, B and C in the Gammaproteobacteria (Figure 2). Here the number of analyzed taxa was smaller because all three genes were not always successfully amplified by PCR from the louse samples. However, the phylogenetic relationships were entirely concordant with the analysis of the 16S rRNA sequence data alone (Figure 1). Notably, both ML and Bayesian support values were higher for the three clades of *Columbicola* spp. symbionts in the combined tree in comparison to the tree derived from 16S rDNA alone. This reflects the fact that the protein-coding sequences evolve in a more stable manner, allowing us to generate an alignment with less ambiguity with respect to outgroups.

### Star-like phylogeny in the clade A of *Columbicola* spp. symbionts

In the 16S rRNA gene phylogeny (Figure 1), the symbiont sequences from different individuals of the same species/cryptic species/haplogroups formed monophyletic groups with high statistical support. In many cases these sequences were identical. However, deeper relationships between the symbionts of *Columbicola* spp. were not well resolved regardless of the reconstruction method employed. In particular, the internodes connecting the representatives of clade A were extremely short with little or no statistical support, although substantial sequence divergence was observed among the representatives of clade A, as evidenced by the relatively long branches leading to terminal nodes. In clade A, consequently, the phylogeny exhibited a comb- or star-like appearance, except for the following statistically-supported terminal clusters of recent origin: (i) *C. fortis* #16 and *C. rodmani #*38; (ii) *C. adamsi* #1 and *C. waggermani* #43; and (iii) *C. columbae* #9, 10 and *C. tschulyshman* #41 (Figure 1). In the phylogeny based on the combined 16S rRNA, *fusA* and *groEL* gene sequences (Figure 2), no clusters with significant statistical support were identified in clade A, which consistently contained long terminal branches and corroborated the star-like phylogenetic relationship in the clade A symbionts of *Columbicola* spp.

### Lack of host-symbiont phylogenetic congruence

Using the Shimodaira and Hasegawa (S-H) test [20], the phylogenetic tree of *Columbicola* spp. [21] was found to be significantly different to the symbiont 16S rRNA tree (difference in ln L = 575.24, *P* < 0.001) and the symbiont tree derived from the combined dataset (difference in ln L = 46.43, *P* < 0.001).

**Figure 3:**
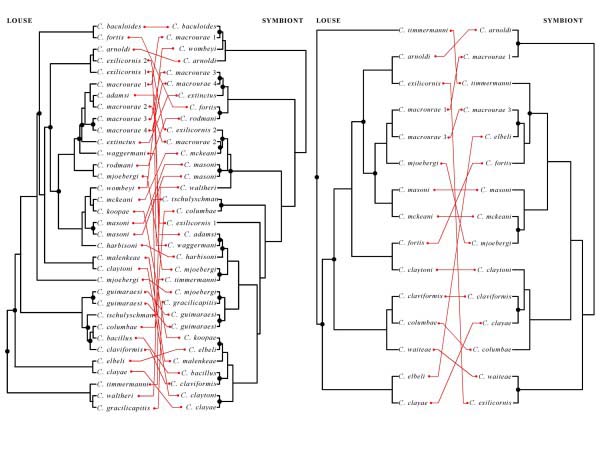
**Comparison of the phylogenies of representative species of *Columbicola *spp. and their symbiotic bacteria.***Columbicola* trees are from maximum likelihood analysis of sequences of the mitochondrial cytochrome oxidase I gene, mitochondrial 12S rRNA gene, and nuclear elongation factor 1alpha gene [21]. Symbiont trees are from Figure 1 (left) and Figure 2 (right) in the current paper. Connecting lines illustrate host-symbiont associations. Bulleted nodes are co-speciation events inferred from reconciliation analysis [22].

Co-phylogenetic analysis [22] of the 16S rRNA gene dataset reconstructed 17 potential co-speciation events between the host and symbiont lineages (Figure 3). However, this number of co-speciation events was not significantly higher than that expected by chance (*P* > 0.05). Using the combined dataset of 16S rRNA, *fusA* and *groEL* genes, co-phylogenetic analysis reconstructed only 6 potential co-speciation events (Figure 3), again no more than that expected by chance (*P* > 0.05).

### Molecular evolutionary rate estimations

Relative rate tests revealed that the substitution rates in 16S rRNA genes of the clade A (*C. columbae*), clade B (*C. baculoides*), and clade C (*C. arnoldi*) symbionts were significantly higher than those of their free-living relatives. Clade B and clade C symbionts exhibited 1.42 fold and 1.35 fold higher rates, respectively, which were supported by *P*-values < 0.001, while the clade A symbiont showed a 1.17 fold higher rate, which was supported by a *P*-value < 0.01 (Table 1).

**Table 1:** **Relative-rate tests comparing molecular evolutionary rates of 16S rRNA gene sequences between different lineages of the symbionts of *Columbicola *** **spp. and free-living relatives**

**Lineage 1**	**Lineage 2**	**Outgroup**	**K1**^**1**^	**K2**^**2**^	**K1-K2**	**K1/K2**	***P-*** **value**^**3**^
Symbiont of *C. columbae* (clade A) [JQ063426]	*Dickeya dadantii* [AY360397]	*Vibrio cholerae* [CP001486.1]	0.124	0.106	0.018	1.170	0.012
Symbiont of *C. baculoides* (clade B) [JQ063412]	*Salmonella enterica* [AP011957]	*Vibrio cholerae* [CP001486.1]	0.143	0.101	0.042	1.416	2.19 × 10^-5^
Symbiont of *C. arnoldi* (clade C) [JQ963434]	*Escherichia coli* [U00096]	*Vibrio cholerae* [CP001486.1]	0.142	0.105	0.037	1.352	9.18 × 10^-6^
Symbiont of *C. columbae* (clade A) [JQ063426]	Symbiont of *C. baculoides* (clade B) [JQ063412]	*Vibrio cholerae* [CP001486.1]	0.124	0.143	−0.019	0.867	0.105
Symbiont of *C. baculoides* (clade B) [JQ063412]	Symbiont of *C. arnoldi* (clade C) [JQ963434]	*Vibrio cholerae* [CP001486.1]	0.143	0.142	0.001	1.007	0.981
Symbiont of *C. arnoldi* (clade C) [JQ963434]	Symbiont of *C. columbae* (clade A) [JQ063426]	*Vibrio cholerae* [CP001486.1]	0.142	0.124	0.018	1.145	0.065
Symbiont of *C. columbae* (clade A) [JQ063426]	*Sodalis glossinidius* (clade A) [AF548135]	*Vibrio cholerae* [CP001486.1]	0.124	0.106	0.018	0.169	0.001
*Sodalis glossinidius* (clade A) [AF548135]	*Dickeya dadantii* [AY360397]	*Vibrio cholerae* [CP001486.1]	0.106	0.106	0	0	0.998

^1^Estimated mean distance between lineage 1 and the last common ancestor of lineages 1 and 2.

^2^Estimated mean distance between lineage 2 and the last common ancestor of lineages 1 and 2.

^3^*P*-value was generated using the program RRTree [23].

### In vivo localization of *C. baculoides* symbiont

**Figure 4:**
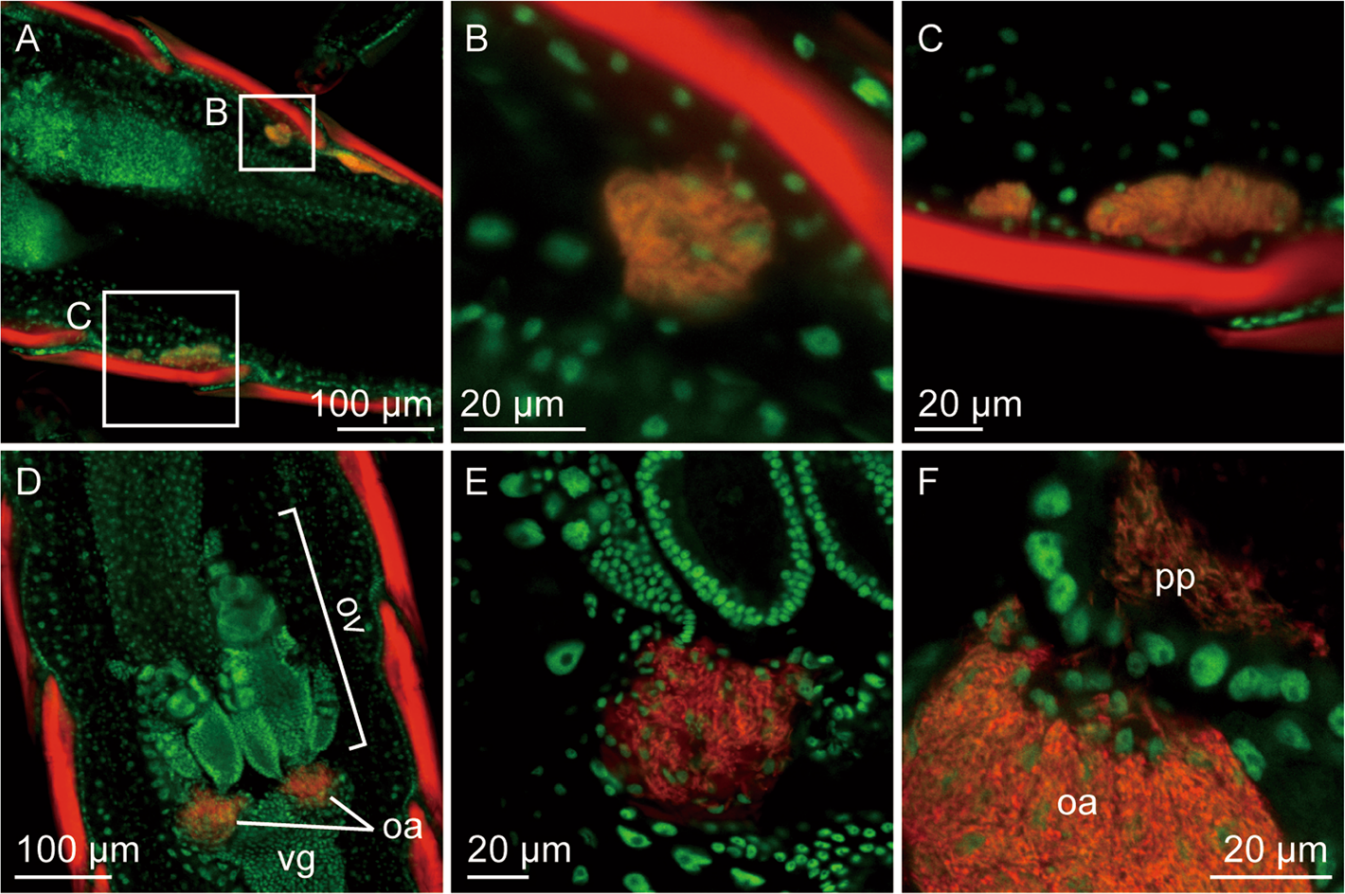
**Fluorescent *in situ *hybridization of *C. baculoides symbiont cells.*** (**A**) Abdominal image of an adult male. Signals of the symbiont cells are detected in bacteriocyte clusters located on both sides of the abdominal body cavity. White squares indicate the areas of panels **B** and **C**. (**B**, **C**) Enlarged images of the bacteriocyte clusters in panel **A**. Signals of the symbiont cells are localized in the cytoplasm of the bacteriocytes. (**D**) Abdominal image of an adult female. Signals of the symbiont cells are localized in ovarial ampullae located at the base of the ovaries. (**E**) An enlarged image of an ovarial ampulla. (**F**) A snapshot of symbiont transmission from an ovarial ampulla to a developing oocyte. Red and green signals indicate symbiont 16S rRNA and host nuclear DNA, respectively. Abbreviations: oa: ovarial ampulla, ov: ovariol, vg: vagina, pp: posterior pole of oocyte.

In males of *C. baculoides*, fluorescent *in situ* hybridization detected the symbiont cells within bacteriocytes that clustered on both sides of the abdominal cavity (Figure 4A, B and C). In young females of *C. baculoides*, the symbiont cells exhibited the same localization as in males (not shown). In mature females, by contrast, the symbiont cells were found in ovarial tissues, localized in a pair of specialized transmission organs called ovarial ampullae (Figure 4D and E), and vertically transmitted from the ovarial ampullae to the posterior pole of developing oocytes (Figure 4F). The localization, migration and transmission patterns of the clade B symbiont of *C. baculoides* are almost identical to those of the clade A symbiont of *C. columbae*[13].

### Simulating symbiont replacements

**Figure 5:**
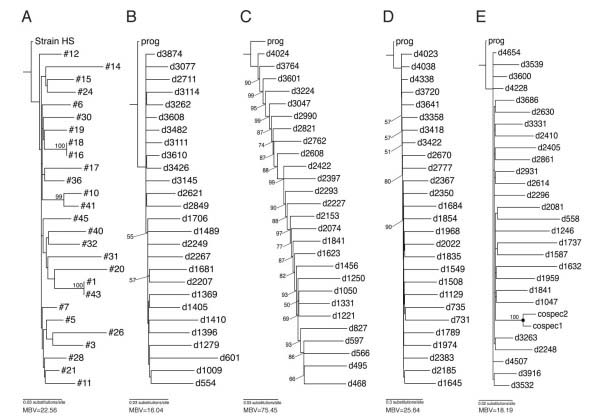
**Bootstrapped ML trees derived from a reduced clade A dataset and sequences derived from evolutionary simulations.** The tree depicting the reduced clade A dataset (panel **A**) comprises only strain HS and *Columbicola* spp. symbionts (with terminal nodes labeled according to the numbers listed in Additional File 5). Trees in panels **B** – **E** are derived from the output generated by simulation with evolutionary rates (substitutions/site/cycle) of 0.002 (progenitor) and 0.016 (descendants) for panel **B** and **E** , 0.016 for both the progenitor and descendants for panel **C** and 0.016 (progenitor) and 0.128 (descendants) for panel **D**. Terminal nodes representing the progenitor sequence are labeled “prog” and descendants are labeled with the prefix “d”, followed by the cycle number of their birth in the 5000 cycle simulation. The tree in panel **E** was obtained from a dataset in which one descendant was permitted to speciate at cycle 2500, giving rise to “cospec1” and “cospec2”. Mean bootstrap values (MBV), depicted at the foot of each tree were computed from bootstrap values obtained for all internal nodes in each tree. Only individual bootstrap values > 50% are depicted in the figure.

In common with the complete tree (Figure 1), the 16S rRNA tree comprising only strain HS and the clade A *Columbicola* spp. symbionts also had a comb-like topology with low statistical support (Figure 5A). When the discrete-time Monte Carlo simulation was optimized to yield progenitor and descendant divergence levels matching those obtained from the real data (Figure 5A), the resulting tree (Figure 5B) was found to be very similar to the tree depicted in Figure 5A both in terms of its comb-like topology and low statistical support. In addition, both trees had a relatively wide variance in terminal branch lengths. Since our simulations only introduce point mutations and therefore yield unambiguous sequence alignments, this indicates that the lack of resolution in the real trees is indeed a function of the underlying evolutionary process, rather than an artifact of the tree-building process.

Since the tree presented in Figure 5B was generated from a simulation in which descendants evolved at a rate that was eight fold higher than their progenitor, we were curious to test the effect of increasing the progenitor evolutionary rate such that it matched that of the descendants. Interestingly, when the simulation was performed with a rate of progenitor sequence evolution that equaled that of the descendants, the resulting tree was found to have a significantly increased mean bootstrap support (Figure 5C). While the topology of the tree retained a comb-like appearance, all nodes were resolved with high bootstrap support in accordance with the temporal order of their descent from the progenitor. The overall increase in the level of bootstrap support for the tree in Figure 5C could be due to either (i) a simple increase in the amount of signal in the phylogenetic analysis or (ii) an increase in the ratio of signal to noise arising from an increased progenitor: descendant mutation rate. To resolve this question we performed a third simulation run in which the rate of descendant sequence evolution was elevated a further eight fold in comparison to the rates used in the previous run, yielding the tree presented in Figure 5D. Although the rates of progenitor evolution are identical in the runs yielding Figures 5C and 5D, the mean bootstrap values for the tree presented in Figure 5D are reduced to a level comparable to that observed in Figure 5B. This indicates that, under the scenario of repeated symbiont replacements modeled in our simulation, an increase in the relative rate of evolution of the descendants, as observed in the real data, has a strong negative impact on the ability to resolve relationships between the progenitor and descendants. Finally, we performed an additional simulation run (with parameters identical to those used to generate Figure 5B) in which one descendant speciated midway through the simulation run, to mimic a host-symbiont co-speciation event. Notably, the relationship between the resulting descendants (labeled cospec1 and cospec2 in Figure 5E) was resolved with 100% bootstrap support, indicating that it is the underlying evolutionary process, rather than the data, alignment properties or phylogenetic method that results in low bootstrap support for our comb-like trees. Thus, at a fundamental level, our results show that a relatively unsupported comb-like tree topology is expected under a scenario in which multiple independent symbiont replacements take place in a limited time frame, because descendants have very few synapomorphies that have accrued in the slow-evolving progenitor lineage. However, when the symbiotic descendants co-speciate with their insect host, synapomorphies are seeded in the ancestral symbiotic lineage at an elevated rate, yielding a signal that can be used to infer their phylogenetic relationships.

### Evidence for recent symbiont replacement events in *Columbicola* spp

The pairwise estimates of synonymous sequence divergence for the *fusA* and *groEL* sequences allowed us to estimate the time since divergence of strain HS and the various *Columbicola* spp. clade A symbionts at between 54,000 and 367,000 years (Table 2). In comparison with rates of divergence obtained for some ancient primary insect symbionts found in other insect hosts, the acquisition of the *Columbicola* spp. clade A symbionts seems to be a relatively recent event, that is far younger than the radiation of the majority of *Columbicola* spp.

**Table 2:** **Pairwise estimates of synonymous divergence ( *d *** **S) and estimates of time since divergence (TSD) between strain HS and symbionts of *Columbicola *** **spp., *Sodalis glossinidius *** **and the *Sitophilus oryzae *** **symbiont, SOPE**

	***groEL***		***fusA***	
**Species**	***d*** **S**	**TSD (years)**	***d*** **S**	**TSD (years)**
*C.clayae* (#6)	n/a^1^	n/a^1^	0.428	260,522
*C. mjoebergi* (#33)	n/a^1^	n/a^1^	0.421	256,261
*C. masoni* (#30)	n/a^1^	n/a^1^	0.341	207,565
*C. timmermanni* (#40)	n/a^1^	n/a^1^	0.310	188,696
*C. fortis* (#16)	0.345	214,388	0.273	166,174
*C. mckeani* (#31)	0.394	245,439	0.224	136,348
*C. exilicornis* (#12)	0.117	72,852	0.236	143,652
*C. waiteae*	0.238	148,320	0.201	122,347
*C. columbae* (#10)	0.200	124,725	0.223	135,739
*C. claviformis* (#5)	n/a^1^	n/a^1^	0.136	82,782
*C. claytoni* (#7)	n/a^1^	n/a^1^	0.089	54,173
*C. macrourae* (#26)	0.590	367,213	n/a^1^	n/a^1^
*C. elbeli* (#11)	0.542	337,209	n/a^1^	n/a^1^
*S. glossinidius*	0.245	152,528	0.163	99,217
SOPE	0.045	28,000	0.046	28,000

^1^Symbiont sequence not available for pairwise comparison. See Additional file 5.

## Discussion

We undertook an extensive molecular survey of bacterial symbionts associated with feather-feeding lice of the genus *Columbicola*, which are found on most species of pigeons and doves across the globe. The molecular phylogenetic analyses grouped the symbionts of *Columbicola* spp. into three putative clades, designated A, B and C, in the Gammaproteobacteria, indicating polyphyletic evolutionary origins of these symbionts. While statistical support for the monophyly of the three clades was only marginal in the case of the 16S rRNA tree, stronger support was observed in the case of the combined tree, which incorporated data from protein-coding genes. Each representative louse sample screened in our study was associated with a single bacterial symbiont derived from one of the three clades. The same pattern was consistently observed in multiple individuals of the same louse species and in cryptic species, indicating that there is some level of stability in the louse-symbiont associations. Based on these results, we conclude that each of the columbiform feather lice of the genus *Columbicola* is associated with a single primary bacterial symbiont, while the symbionts associated with different species of *Columbicola* are phylogenetically diverse.

In many obligate host-symbiont associations, such as those observed between aphids and *Buchnera aphidicola*, and tsetse flies and *Wigglesworthia glossinidia*, a specific host taxon is known to have adopted a single symbiont that is maintained over macroevolutionary time through repeated bouts of co-speciation, yielding congruent host and symbiont phylogenies [3,24]. In this study we demonstrated that evolutionary patterns of host-symbiont association are distinct in the *Columbicola* feather lice: the primary symbionts are of polyphyletic evolutionary origins and do not exhibit any significant degree of host-symbiont co-speciation. Similar polyphyletic primary symbionts have been reported from weevils of the subfamily Dryophthorinae [25,26], and sucking lice of the suborder Anoplura [27]. However, the case of *Columbicola* spp. is remarkable in that these polyphyletic primary symbionts are found within a single genus of host insects.

Relative rate tests revealed that molecular evolutionary rates are elevated in representatives of all three symbiont clades associated with *Columbicola* spp., but the levels of acceleration are more pronounced in clades B and C relative to clade A (Figure 1, Table 1). This pattern suggest that clade B and clade C may represent more ancient symbiont lineages that have experienced a longer history of host-symbiont co-evolution, with an associated accelerated evolutionary rate. This in turn implies that representatives of clade A have a more recent origin of symbiosis. The different symbiont lineages may have been acquired by the different *Columbicola* lineages independently or, alternatively, representatives of clade A may have replaced the putatively more ancient clade B and C lineages. The replacement scenario seems more likely, given that all *Columbicola* species examined in this study were found to harbor only a single symbiont, and that representatives of both clade A and B exhibit the same localization, migration and transmission patterns according to FISH-based microscopic analyses (Figure 4) [13].

Previous studies suggested that *Columbicola* feather lice diversified mainly in the Paleogene [28,29]. Thus, if replacements have occurred, they must have occurred since this time. Similar symbiont replacements have been reported from weevils of the family Dryophthoridae, where the ancient symbiont lineage *Nardonella* was replaced by several symbiont lineages that are predicted to be of more recent origin [25,26]. Indeed, replacements involving *Sitophilus* spp. and their clade A symbionts, SOPE and SZPE, are now predicted to have taken place very recently (<28,000 years), based on analysis of genome-wide substitution rates between SOPE and strain HS [17]. Also, in aphids of the tribe Cerataphidini, the ancient symbiont *Buchnera aphidicola* is thought to have been replaced by fungal symbiont lineages [30-32]. In the case of the *Columbicola* spp. clade A symbionts it is important to note that our estimates of time since divergence from strain HS indicate an extremely recent origin for these symbiotic associations (<0.4 MY; Table 2). Since the radiation of the *Columbicola* spp. complex is estimated to have encompassed approximately 57 million years [28,29], we predict that there has been little opportunity for co-speciation between *Columbicola* spp. and their newly acquired clade A symbionts. Thus, it is not surprising that we found very little evidence of congruence between the phylogenies of *Columbicola* spp. and their clade A symbionts. The only exceptions were the cases of very recent divergence between *C. columbae* and *C. tschulyshman*, both of which occur on Rock Pigeons, and the symbionts of *Columbicola macrourae* 3 (on Mourning Dove) and *C. macrourae* 4 (on Galapagos Dove). These two doves are predicted to have diverged from one other less than 2 million years ago [29], and based on genetic divergences, their lice are predicted to have diverged even more recently (around 0.2 MYA, [21]).

Molecular phylogenetic analyses of feather lice reveal several well-supported clades within the genus *Columbicola*[21]. Thus, it is striking that almost all of the internal branches in clade A are short, generating a comb-like tree topology with very little overall bootstrap support (Figure 1). While the unresolved nature of clade A could be ascribed to artifactual issues related to the alignment of diverse sequences in the trees presented in Figures 1 and 2, reconstruction of the 16S rRNA tree with only the *Columbicola* spp. clade A symbionts (resulting in a relatively unambiguous alignment) did not improve the resolution of the tree (Figure 5A).

In weevils of the Dryophthoridae, it has been suggested that symbiont replacements might have been driven by major changes in the insect diet [25]. In contrast, all *Columbicola* species are obligate parasites of columbiform birds that live on a diet of feathers, secretions and dead skin. Experimental transfers of *Columbicola* spp. between different species of pigeons and doves show that these lice are capable of feeding, surviving and reproducing on heterospecific hosts [33]. Hence, symbiont replacements in *Columbicola* spp. are unlikely to be attributable to dietary changes. Previous studies have suggested that biological vectors such as parasitic wasps and mites might facilitate symbiont transfers and replacements across different host species [34,35]. However, neither parasitoid wasps nor ectoparasitic mites have been reported from *Columbicola* spp. [4]. Another possibility is that horizontal symbiont transfers are mediated between different louse species by interspecific mating, as reported for facultative symbionts in the pea aphid [36]. While there is no evidence of interspecific mating in *Columbicola* spp., it is notable that these lice undergo host switching through phoretic dispersal on hippoboscid louse flies [8]. This could at least facilitate contact between males and females of different louse species. It is also noteworthy that male lice often remain in copula with their female partners for several hours [4], which could provide a window for horizontal (male to female) symbiont transfers.

However, phylogenetic lines of evidence do not favor the above-mentioned hypothesis of horizontal symbiont transfers between *Columbicola* spp. If different *Columbicola* species had been undergoing occasional symbiont transfers, the resulting symbiont phylogenetic tree would be expected to be of compact shape, with relatively short terminal branches, as observed for facultative insect symbionts, such as *Wolbachia*, *Rickettsia*, *Hamiltonella*, *Regiella* and *Serratia*[37-39]. Contrary to this expectation, the phylogenies of the symbionts of *Columbicola* spp. are characterized by long terminal branches and very short internodes, giving the trees their comb- like topologies (Figure 1, Figure 2, Figure 5A).

To account for the tree topologies observed in our study we propose an alternative hypothesis that involves repeated symbiont acquisitions from a common bacterial “progenitor” that is ubiquitous in the environment. Such a candidate was recently isolated from a human wound obtained following impalement with a dead tree branch [17]. The resulting isolate, named “strain HS”, was found to be a member of the *Sodalis*-allied clade of insect symbionts, designated as clade A in the current study (Figure 1, Figure 2). Furthermore, it was shown that the gene inventories of two distinct *Sodalis*-allied symbionts represent reduced subsets of strain HS, supporting the notion that an ancestral relative of strain HS served as a progenitor for these insect associates. This progenitor hypothesis is compatible with the following observations and evolutionary patterns derived from our study: First, the symbionts of *Columbicola* spp., in particular those of the clade A, are closely related to symbionts of phylogenetically distant insect hosts that encompass diverse geographical and ecological habitats, such as tsetse flies, louse flies, grain weevils, chestnut weevils, longicorn beetles and stinkbugs [40-47]. Second, the symbiont lineages of *Columbicola* spp., tsetse flies and grain weevils, which show ~100% infection frequencies [13,40,41], suggestive of relative stability and continuity of the associations, are generally characterized by long terminal branches in the phylogeny (Figure 1). Third, in contrast, strain HS and the symbiont lineages of chestnut weevils and stinkbugs, which show low infection frequencies [45-47], which may be indicative of instability and/or a temporary nature of these associations, are characterized by very short terminal branches in the phylogeny (Figure 1). Thus, we postulate that the clade A symbionts of *Columbicola* spp. were acquired from a progenitor lineage that persists in the environment with a low rate of molecular evolution, and that establishment of the vertically-transmitted endosymbiotic lifestyle results in accelerated molecular evolution of the symbiont genes [48,49], giving rise to the long terminal branches observed in our phylogenies. Based on these assumptions, older *Columbicola* symbiont lineages are anticipated to have experienced accelerated molecular evolution for longer periods of time and are thus expected to exhibit longer terminal branches, whereas recent symbiont lineages are expected to have experienced reduced evolutionary acceleration and thus exhibit shorter terminal branches (Figure 5).

In order to determine how a process of recent symbiont acquisition might influence the reconstruction of phylogenetic trees encompassing such events, we developed an *in silico* simulation of this evolutionary process, parameterized using metaheuristic approaches that allow the simulation to closely approximate sequence divergences observed between strain HS and the *Columbicola* spp. clade A symbionts. Data obtained from the simulation yielded a tree (Figure 5B) that is strikingly similar to the tree obtained from the real clade A dataset (Figure 5A). This indicates that a process of symbiont acquisition (or replacement) in which a slowly-evolving progenitor gives rise to more rapidly evolving symbiotic descendants is expected to yield a tree with a comb-like topology that has little statistical support. Further simulation runs with elevated evolutionary rates demonstrated that low levels of bootstrap support are obtained when the rate of evolution of the symbiont sequences is increased relative to the progenitor, However, when a symbiont was permitted to speciate in the simulation, mimicking host-symbiont co-speciation and facilitating synapomorphies in the resulting lineages, the resulting node was resolved with a high level of bootstrap support. Thus, we conclude that the comb-like topology and low statistical support observed in clade A is an anticipated outcome of an underlying evolutionary process in which repeated symbiont replacement or acquisition events occur in a short time period. We also infer that the few highly supported nodes in the *Columbicola* spp. clade A symbionts reflect very recent host-symbiont co-speciation events.

## Conclusions

In conclusion, we have demonstrated unexpected diversity and evolutionary dynamics of the bacterial symbionts in feather lice of the genus *Columbicola*. To account for the peculiar evolutionary patterns observed in *Columbicola-*symbiont associations, we propose a hypothesis of repeated, recent symbiont acquisition/replacement events from a common environmental “progenitor” lineage such as the recently discovered “strain HS” [17]. Using a simulation-based approach, we show that a process of symbiont replacement leads to a characteristic comb-like topology in phylogenetic trees derived from the symbionts. The polyphyletic bacterial symbionts of *Columbicola* spp. highlight the diversity and complexity of insect-microbe symbiotic systems, and provide insights into how such symbiotic associations have established and diversified in nature.

## Methods

### Sample collection and DNA sequencing

Samples of lice were collected from wild birds using the post mortem ruffling procedure [50]. DNA was extracted from individual louse samples by first removing the head and placing both the head and abdomen in extraction buffer ATL (Qiagen). DNA was then isolated using a Qiagen DNAeasy Tissue Extraction Kit. Prior to DNA extraction, body parts were removed and mounted in balsam on microscope slides as morphological vouchers.

DNA extracts were used as template for PCR amplification of a segment of the bacterial 16S rRNA gene (1.46-kb). For a subset of our samples we also amplified a 0.76-kb fragment of the elongation factor EF-G (*fusA*) gene and a 1.49kb fragment of the heat shock chaperone (*groEL*) gene. We used the universal primers 27F (5’- AGAGTTTGATCCTGGCTCAG-3’) and 1492R (5’-TACGGTTACCTTGTTACGACTT-3’) to amplify 16S rRNA gene, the degenerate primers GroELF1 (5’ – ATGGGCWGCWAAAGAYGTRAAAT – 3’) and GroELR1 (5’ – TCGGTRGTGATMATCAGRCCRGC-3’) (designed from an alignment of insect symbionts and other free living members of the Gammaproteobacteria) to amplify the *groEL* gene, and FusAF (5’-CAT CGG CAT CAT GGC NCA YAT HGA-3’) and FusAR (5’-CAG CAT CGG CTG CAY NCC YTT RTT-3’) [51] to amplify the *fusA* gene.

The PCR products were purified using the Qiagen gel extraction kit and concentrated in Microcon columns (Millipore). Purified products were cloned into a TOPO 2.1 vector (Invitrogen). Sanger sequencing reactions were performed on 48 clones derived from *C. columbae* and *C. baculoides* DNA, and a minimum of four clones derived from each of the other DNA samples. Sequences were resolved and checked in the software package Lasergene (DNAStar, Inc. Madison, Wisconsin). All symbiont sequences were deposited in the DDBJ/EMBL/GenBank nucleotide sequence databases under the accession numbers listed in Additional file 5.

### Molecular phylogenetic analyses

In order to reconstruct the phylogeny of the *Columbicola* spp. symbionts, MUSCLE [52] was used to align sequences of the 16S rRNA gene alone, and a combined dataset comprising 16S rRNA, *fusA* and *groEL* genes. The alignments were inspected and adjusted manually and are available upon request from K.F.O. Sequences from other symbionts and free-living bacteria were selected for inclusion on the basis of sequence similarity, using the BLAST search tool (NCBI). We used this approach to ensure that, for each *Columbicola* spp. symbiont sequence, the three most closely related symbiont sequences and the most closely related free-living bacterial sequence from GenBank were represented in our dataset. The remaining free-living taxa were selected to provide appropriate resolution within the family Enterobacteriaceae (Additional file 6). *Vibrio cholerae* was selected as an outgroup because it represents a distantly related member of the family Enterobacteriaceae. We then used Modeltest [53] and JModelTest [54] for Bayesian Information Criteria to infer the most appropriate model of sequence evolution (GTR + I + G) for subsequent analyses. Analysis of 16S rRNA gene sequences was performed using the maximum likelihood (ML) approach implemented in PhyML [55], with 25 random starting trees and 100 bootstrap replicates. Bayesian posterior probabilities were estimated using MrBayes 3.1.2 [56]. Runs were carried out for 1.5 million generations using the default parameters of 4 chains (3 heated and one cold) and stopped when the standard deviation of split frequencies converged to less than 0.00001. The first 4000 generations were discarded as burn-in based on the stabilization of log likelihood values at this point. Consensus trees were built based on the 50% majority rule consensus. For the combined analysis, we first used a partition homogeneity test [57-59] to test for conflict between the three sequences in our combined dataset (16S rRNA, *fusA* and *groEL* genes). Since there were several taxa from which we could only obtain sequence data for two of the three loci, absent sequences were treated as missing data in the tree-building software. For the combined analysis, data were partitioned and parameters were estimated separately for each gene.

### Analysis of 16S rRNA secondary structure

In order to determine the impact of substitutions in the *Columbicola* spp. symbiont sequences on the structure of their 16S rRNA molecules, we mapped substitutions from these sequences onto the secondary structure of the 16S rRNA sequence derived from the most closely related outgroup (*Yersinia pestis*) for which a secondary structure has been deduced [60]. The resulting homology models were then used to compute the relative ratios of substitutions resulting in (i) changes that preserve the secondary structure of the molecule and (ii) changes that induce perturbations in structure (i.e. stem-loop transitions) [15]. We then further analyzed the positions of substitutions in the *Columbicola* spp. symbiont 16S rRNA sequences in accordance with a position-specific variability map computed previously using 3,407 bacterial 16S rRNA sequences [16]. In our analysis, substitutions were scored according to a binary scheme [15], in which sites are designated as having substitution rates that are either higher or lower than the average for all sites analyzed in the variability map study [16].

### Fluorescent in situ hybridization

Oligonucleotide probes specific to the 16S rRNA sequence from *C. baculoides* (5’-GTTTTCTGTTACCGTTCGATT-3’ and 5’-TTGCTTTTTCCTTCTTACTG-3’) were used for whole-mount fluorescent *in situ* hybridization as described previously [13]. Insects obtained from a colony of *C. baculoides* maintained on captive mourning doves (*Zenaida macroura*) were fixed in Carnoy’s solution for two days. The lice were then washed three times in ethanol and immersed in 6% (v/v) H_2_O_2_ in ethanol for 7 d to quench the autofluorescence of insect tissues. After quenching, the insects were washed three times in ethanol and then decapitated and punctured repeatedly with a fine tungsten needle throughout the abdomen. They were then washed twice in ethanol, three times in phosphate-buffered saline containing 0.3% Triton X-100 (PBSTx), and equilibrated in hybridization buffer (20 mM Tris–HCl [pH 8.0], 0.9 M NaCl, 0.01% sodium dodecyl sulfate, 30% formamide). The probes and SYTOX green were added at final concentrations of 100 nM and 0.5 μM, respectively, and the specimens were incubated overnight at room temperature. The specimens were then washed several times in PBSTx, mounted in Slowfade (Invitrogen) and observed under both an epifluorescence microscope (Axiophoto; Zeiss) and a laser confocal microscope (PASCAL5; Zeiss). To confirm specific detection of the symbionts, a series of control experiments were conducted. These consisted of a no-probe control, RNase digestion control, and a competitive-suppression control with excess unlabeled probe as described previously [61].

### Co-phylogenetic analyses

We used two alternative approaches to test for congruence between louse and symbiont phylogenies. First, we conducted Shimodaira-Hasegawa (S-H) tests [20] on the host tree [21] and the ML trees from the 16S rRNA gene alone (Figure 1) and combined sequences (Figure 2) from the symbionts. These trees were pruned to include only a single representative sequence from each louse/symbiont taxon. This method was used to assess whether the symbiont data can be used to reject the louse phylogenetic tree.

As a second method, we reconstructed the number of co-speciation events between the symbiont and louse trees using reconciliation analysis [62] as implemented in TreeMap 1 [22]. This analysis was again performed using both the 16S rRNA gene tree (Figure 1) and the combined tree (Figure 2). As in the S-H test analysis, both the louse and symbiont trees were pruned to include only a single representative of each louse/symbiont taxon to avoid artificially biasing the results in favor of congruence. The symbiont phylogeny was randomized 10,000 times to determine if the number of inferred co-speciation events was greater than that expected by chance [22,63].

### Evolutionary simulation

We used a discrete-time Monte Carlo simulation to model the evolutionary scenario of repeated symbiont replacement by an environmental progenitor. In the simulation, the 16S rRNA sequence of strain HS was used as a candidate progenitor sequence and permitted to accumulate random mutation at a user-defined rate over the course of 5000 simulation cycles. Over this time, the evolving strain HS sequence gave rise to a user-defined number of descendant sequences that subsequently evolve independently at a user-defined rate until the 5000 simulation cycles are complete. The timing of descendant birth is governed by a 2-parameter Beta distribution, with values of α = β = 2, which were stochastically optimized along with the mutation rates of the progenitor and descendants to yield an average pairwise distance and variance between the progenitor and descendant sequences that closely matches the mean pairwise distance and variance estimated from pairwise comparisons between the 16S rRNA sequences of strain HS and the *Columbicola* spp. symbionts of clade A. Sequences obtained at the end of each simulation run were aligned unambiguously and used for the construction of phylogenetic trees, using the same methods as for the construction of the tree in Figure 1. For comparison, an additional tree was also constructed for the 16S rRNA sequences of strain HS and the clade A symbionts of *Columbicola* spp. alone.

### Estimating divergence times between strain HS and other members of clade A

Based on genome-wide estimates of synonymous substitution rates, the divergence between strain HS and the weevil symbiont, SOPE, was predicted to have taken place around 28,000 years ago (17). To estimate the dates of sequence divergence between strain HS and other clade A symbionts we computed pairwise synonymous site divergences for the *fusA* and *groEL* sequences listed in Additional files 5 and 6 using the Kumar method implemented in MEGA [64]. The resulting estimates of sequence divergence were then used to obtain dates of divergence based on extrapolation from the strain HS-SOPE comparisons.

## Abbreviations

ML: Maximum likelihood.

## Competing interests

The authors declare that they have no competing interests.

## Authors’ contributions

DHC collected many of the lice used in the study. WAS, KFO, KPJ, DLR, TF, DHC and CD conceived and designed the study. WAS, DLR, TC and KLS performed molecular genetic experiments leading to sequence collection. WAS, KPJ and KFO performed phylogenetic analyses, and CD performed evolutionary simulations. RK and TF performed *in vivo* localization of the *C. baculoides* symbiont. All authors were involved in drafting the manuscript and have read and approved the final version.

## Supplementary Material

Additional file 1 **Phylogeny of *Columbicola *spp. symbionts and related bacteria based on a 1.46-kb fragment of 16S rRNA.** Insect symbionts are designed by the prefix “PE” (primary endosymbiont), “SE” (secondary endosymbiont) or E (if unknown), followed by host name and common name (for those not derived from *Columbicola* spp.) The numbers adjacent to nodes indicate maximum likelihood bootstrap values (above the line) and Bayesian posterior probabilities, where applicable (below the line), for nodes with bootstrap support >50% and Bayesian posterior probabilities >0.5. Asterisks indicate nodes with 100 % bootstrap support and Bayesian posterior probability = 1. The bold arrow highlights the location of the sequence derived from strain HS, the recently characterized progenitor of the *Sodalis*-allied symbionts. Numbers in parentheses represent the G + C content of the 16S rRNA sequences. Final numbers correspond to the list provided in Supplementary Table 1. Click here for file

Additional file 2 **Homology model depicting the *C. veigasimoni *symbiont 16S rRNA sequence mapped onto the predicted *Y. pestis *16S rRNA structure.** Homology was deduced from an alignment generated in Muscle, and adjusted manually to account for indels. Substitutions in the symbiont 16S rRNA are highlighted in bold. Substitutions with a higher-than-average rate of variability (v > 1) are highlighted with red spots, whereas those with a lower-than-average rate of variability (v < 1) are highlighted with blue spots. The counts of different substitution types are displayed in parentheses in the key. Click here for file

Additional file 3 **Homology model depicting the *C. paradoxus *symbiont 16S rRNA sequence mapped onto the predicted *Y. pestis *16S rRNA structure.** Homology was deduced from an alignment generated in Muscle, and adjusted manually to account for indels. Substitutions in the symbiont 16S rRNA are highlighted in bold. Substitutions with a higher-than-average rate of variability (v > 1) are highlighted with red spots, whereas those with a lower-than-average rate of variability (v < 1) are highlighted with blue spots. The counts of different substitution types are displayed in parentheses in the key. Click here for file

Additional file 4 **Homology model depicting the *C. columbae *symbiont 16S rRNA sequence mapped onto the predicted *Y. pestis *16S rRNA structure.** Homology was deduced from an alignment generated in Muscle, and adjusted manually to account for indels. Substitutions in the symbiont 16S rRNA are highlighted in bold. Substitutions with a higher-than-average rate of variability (v > 1) are highlighted with red spots, whereas those with a lower-than-average rate of variability (v < 1) are highlighted with blue spots. The counts of different substitution types are displayed in parentheses in the key. Click here for file

Additional file 5 **Louse specimens used in the current study.** Information relating to the collection, maintenance and storage of louse specimens used in the current study, along accession numbers of sequences deposited in the Genbank database. Click here for file

Additional file 6 **Additional DNA sequences used in the current study.** Genbank accession numbers of additional sequences used in phylogenetic analyses in the current study. Click here for file
