# Supplementary figures and images for: Phylogenetic analysis of symbionts in feather-feeding lice of the genus Columbicola: evidence for repeated symbiont replacements
Source: BMC Evol Biol. 2013 May 31;13:109. doi: 10.1186/1471-2148-13-109 (PMC3724504; doi:10.1186/1471-2148-13-109)

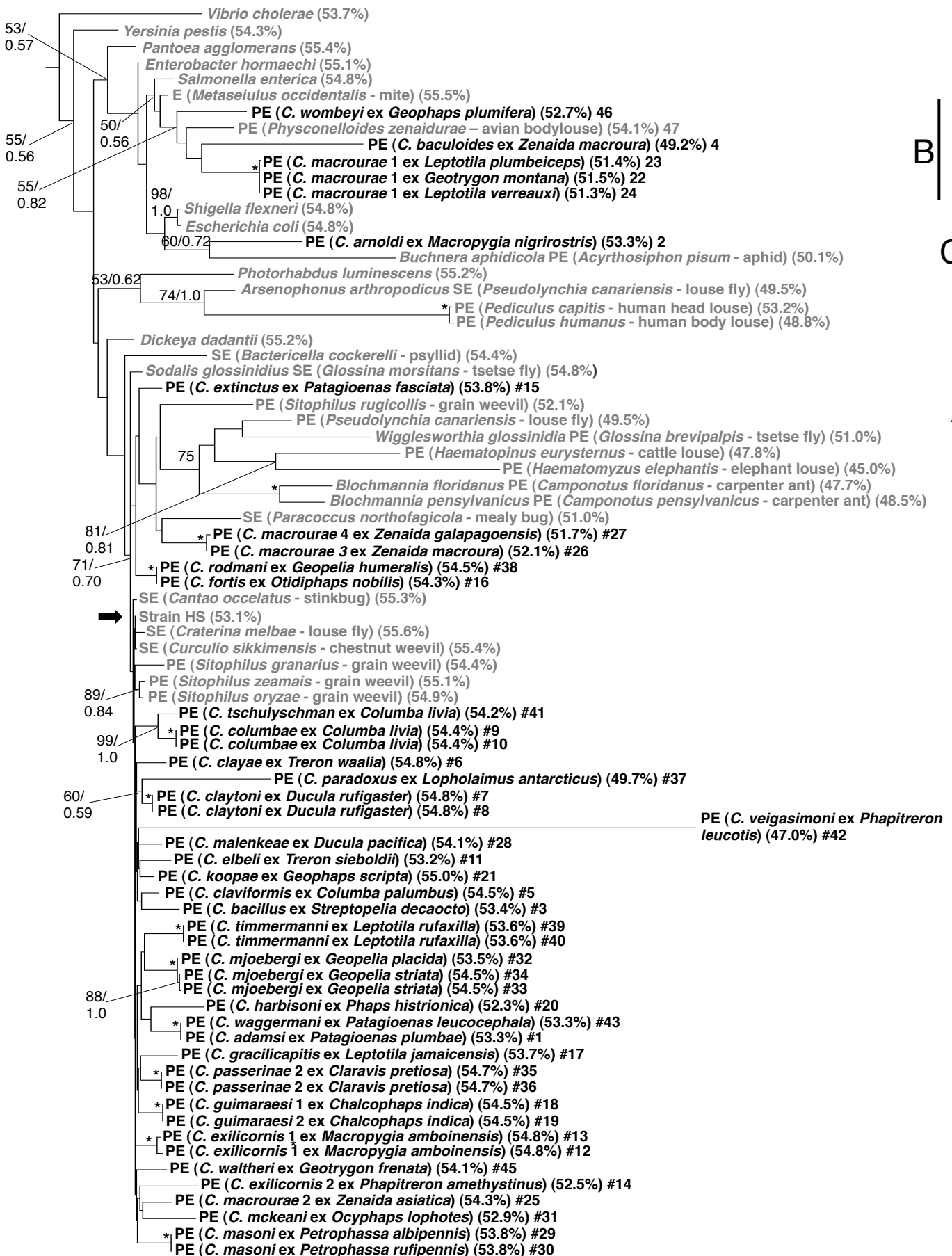

Supplement: Additional file 1 — Phylogeny of Columbicola spp. symbionts and related bacteria based on a 1.46-kb fragment of 16S rRNA. Insect symbionts are designed by the prefix “PE” (primary endosymbiont), “SE” (secondary endosymbiont) or E (if unknown), followed by host name and common name (for those not derived from Columbicola spp.) The numbers adjacent to nodes indicate maximum likelihood bootstrap values (above the line) and Bayesian posterior probabilities, where applicable (below the line), for nodes with bootstrap support >50% and Bayesian posterior probabilities >0.5. Asterisks indicate nodes with 100 % bootstrap support and Bayesian posterior probability = 1. The bold arrow highlights the location of the sequence derived from strain HS, the recently characterized progenitor of the Sodalis-allied symbionts. Numbers in parentheses represent the G + C content of the 16S rRNA sequences. Final numbers correspond to the list provided in Supplementary Table 1. [file 1471-2148-13-109-S1.pdf]

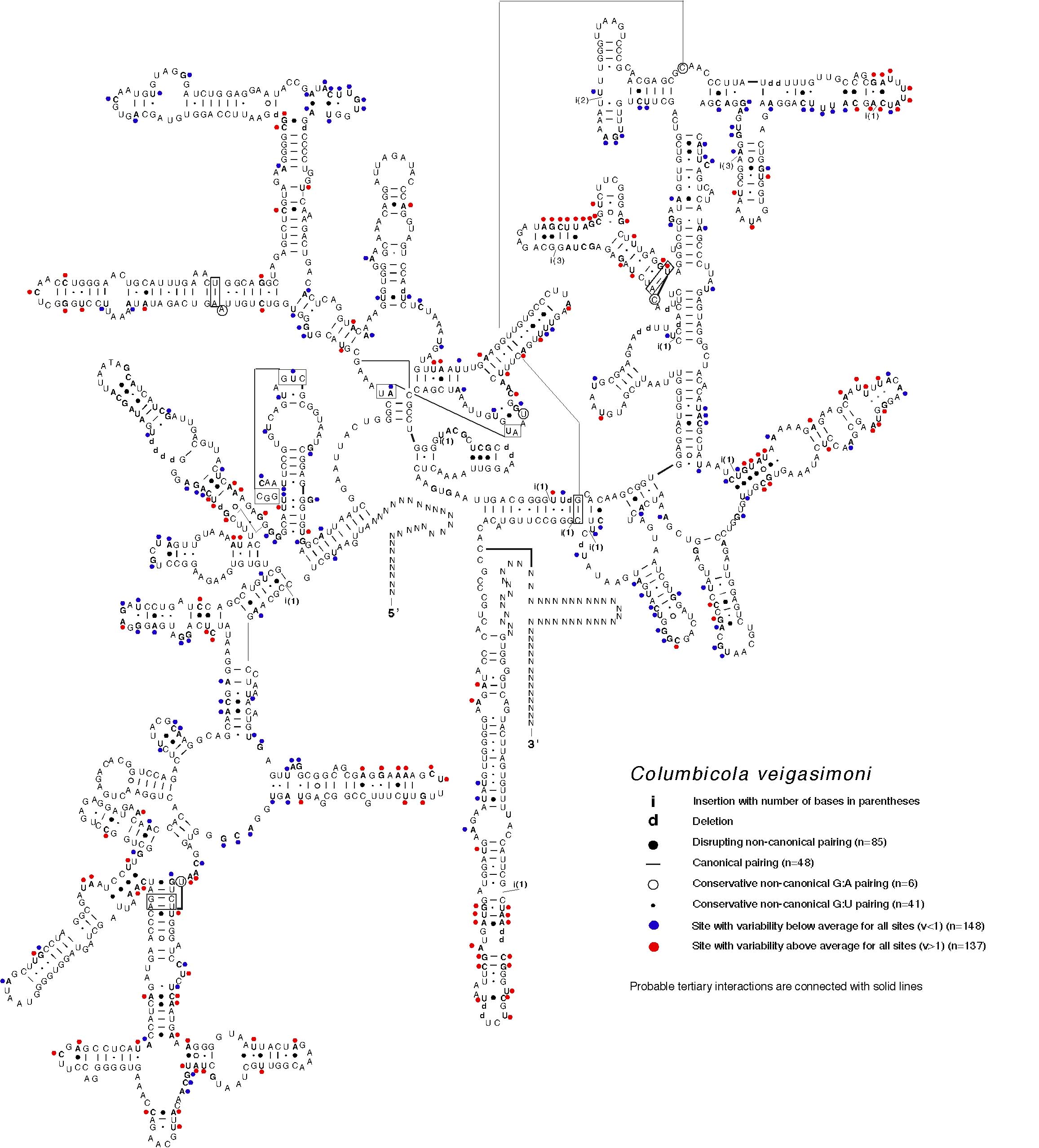

Supplement: Additional file 2 — Homology model depicting the C. veigasimoni symbiont 16S rRNA sequence mapped onto the predicted Y. pestis 16S rRNA structure. Homology was deduced from an alignment generated in Muscle, and adjusted manually to account for indels. Substitutions in the symbiont 16S rRNA are highlighted in bold. Substitutions with a higher-than-average rate of variability (v > 1) are highlighted with red spots, whereas those with a lower-than-average rate of variability (v < 1) are highlighted with blue spots. The counts of different substitution types are displayed in parentheses in the key. [file 1471-2148-13-109-S2.jpeg]

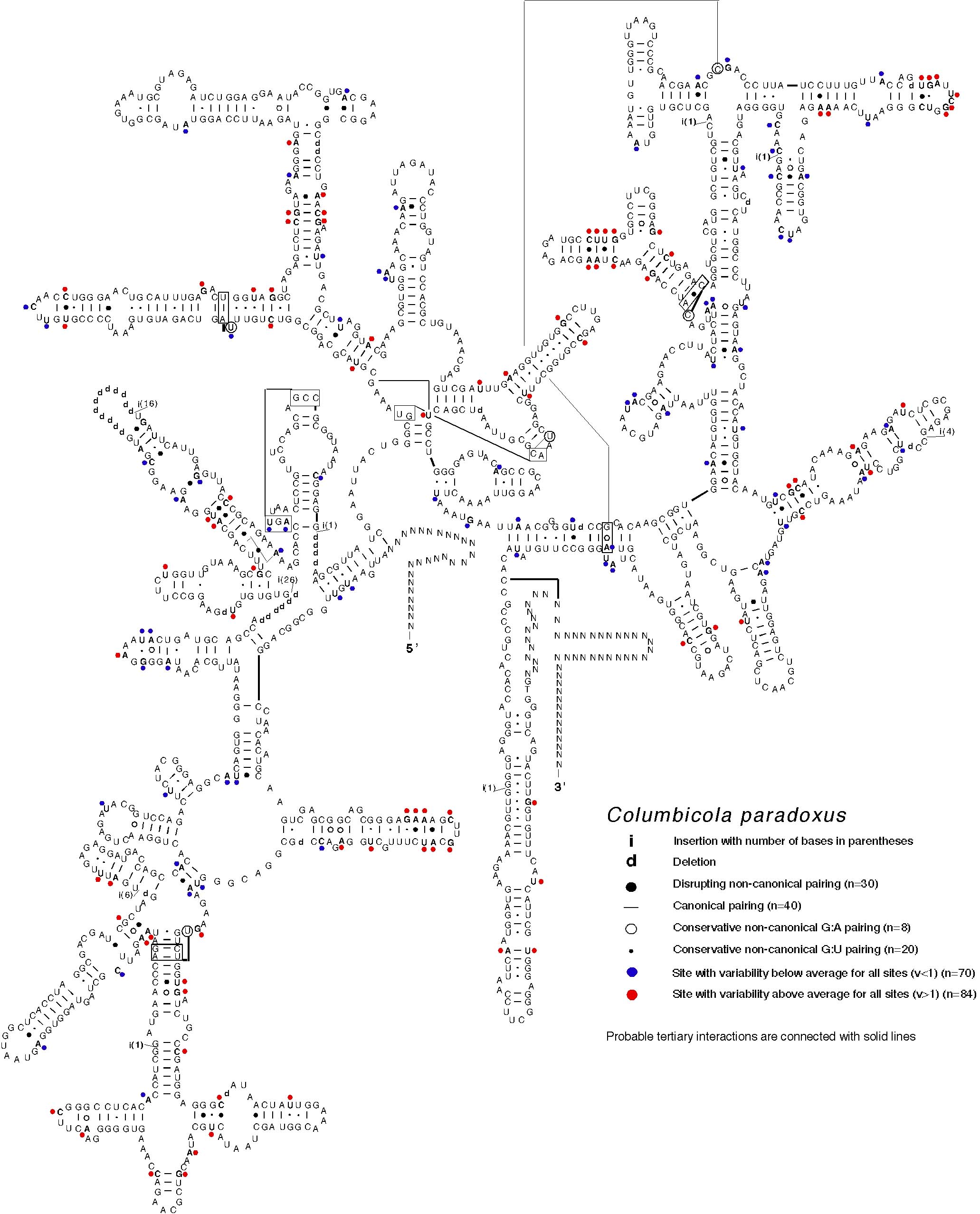

Supplement: Additional file 3 — Homology model depicting the C. paradoxus symbiont 16S rRNA sequence mapped onto the predicted Y. pestis 16S rRNA structure. Homology was deduced from an alignment generated in Muscle, and adjusted manually to account for indels. Substitutions in the symbiont 16S rRNA are highlighted in bold. Substitutions with a higher-than-average rate of variability (v > 1) are highlighted with red spots, whereas those with a lower-than-average rate of variability (v < 1) are highlighted with blue spots. The counts of different substitution types are displayed in parentheses in the key. [file 1471-2148-13-109-S3.jpeg]

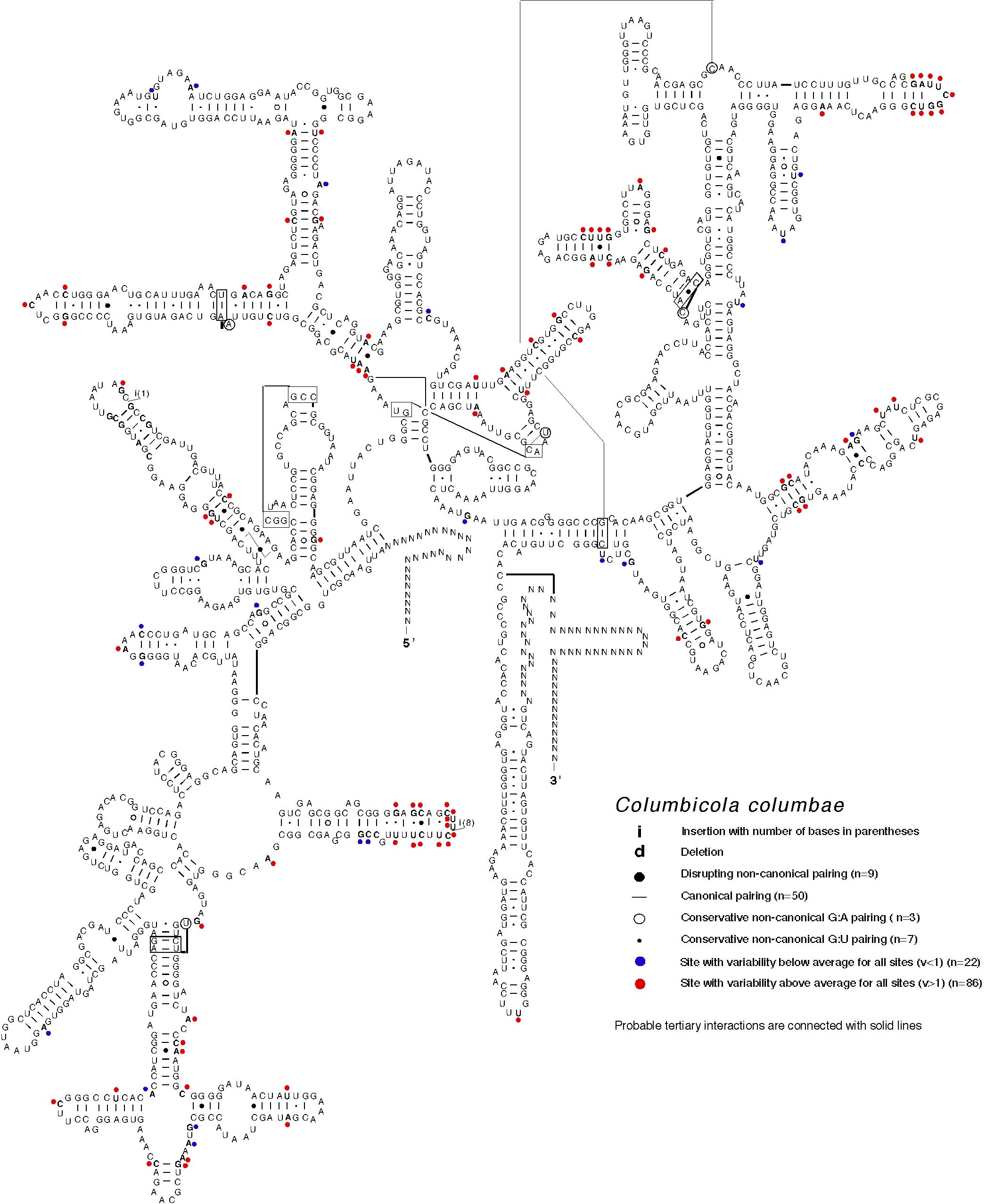

Supplement: Additional file 4 — Homology model depicting the C. columbae symbiont 16S rRNA sequence mapped onto the predicted Y. pestis 16S rRNA structure. Homology was deduced from an alignment generated in Muscle, and adjusted manually to account for indels. Substitutions in the symbiont 16S rRNA are highlighted in bold. Substitutions with a higher-than-average rate of variability (v > 1) are highlighted with red spots, whereas those with a lower-than-average rate of variability (v < 1) are highlighted with blue spots. The counts of different substitution types are displayed in parentheses in the key. [file 1471-2148-13-109-S4.jpeg]
