# Supplementary material for: Phylogenetic analysis of symbionts in feather-feeding lice of the genus Columbicola: evidence for repeated symbiont replacements
Source: BMC Evol Biol. 2013 May 31;13:109. doi: 10.1186/1471-2148-13-109 (PMC3724504; doi:10.1186/1471-2148-13-109)
Supplement: Additional file 5 — Louse specimens used in the current study. Information relating to the collection, maintenance and storage of louse specimens used in the current study, along accession numbers of sequences deposited in the Genbank database. [file 1471-2148-13-109-S5.pdf]

#	Louse	Host	Location	Louse Voucher Code	Gene	GenBank Accession
1	*C. adamsi*	*Patagioenas plumbea*	Guyana	04.24.99.03	16S rDNA	JQ063407
2	*C. arnoldi*	*Macropygia nigrirostris*	Papua New Guinea	05.14.03.05	16S rDNA	JQ963434
					GroEL	JQ063386
3	*C. bacillus*	*Streptopelia decaocto*	Netherlands	11.15.99.01	16S rDNA	JQ063440
4	*C. baculoides*	*Zenaida macroura*	USA	10.19.98.01	16S rDNA	JQ063412
5	*C. claviformis*	*Columba palumbus*	UK	01.20.03.16	16S rDNA	JQ063427
					FusA	JQ063396
6	*C. clayae*	*Treron waalia*	Ghana	03.21.00.09	16S rDNA	JQ063451
					FusA	JQ063405
7	*C. claytoni*	*Ducula rufigaster*	Papua New Guinea	07.26.04.06	16S rDNA	JQ063433
8	*C. claytoni*	*Ducula rufigaster*	Papua New Guinea	08.19.03.14	16S rDNA	JQ063430
					FusA	JQ063395
9	*C. columbae*	*Columba livia*	USA	06.29.98.3	16S rDNA	JQ063426
					FusA	JQ063397
					GroEL	JQ063388
10	*C. columbae*	*Columba livia*	Australia	07.15.02	16S rDNA	JQ063439
11	*C. elbeli*	*Treron sieboldii*	China	06.06.05.04	16S rDNA	JQ063437
					GroEL	JQ063392

12	*C. exilicornis* 1	*Macropygia amboinensis*	Papua New Guinea	08.19.03.07	16S rDNA	JQ063414
					FusA	JQ063398
					GroEL	JQ063387
13	*C. exilicornis* 1	*Macropygia amboinensis*	Papua New Guinea	08.19.0308	16S rDNA	JQ063449
14	*C. exilicornis* 2	*Phapitreron amethystinus*	Phillipines	05.26.99.06	16S rDNA	JQ063408
15	*C. extinctus*	*Patagioenas fasciata*	USA	01.20.03.01	16S rDNA	JQ063416
16	*C. fortis*	*Otidiphaps nobilis*	Papua New Guinea	05.14.03.07	16SrDNA	JQ063452
					FusA	JQ063402
					GroEL	JQ063390
17	*C. gracilicapitis*	*Leptotila jamaicensis*	Mexico	09.29.98.04	16S rDNA	JQ063444
18	*C. guimaraesi* 1	*Chalcophaps indica*	Vanuatu	07.26.04.04	16S rDNA	JQ063431
19	*C. guimaraesi* 2	*Chalcophaps indica*	Australia	07.20.04.12	16S rDNA	JQ063435
20	*C. harbisoni*	*Phaps histrionica*	Australia	05.14.03.09	16S rDNA	JQ063411
21	*C. koopae*	*Geophaps scripta*	Australia	01.08.03.10	16S rDNA	JQ063432
22	*C. macrourae* 1	*Geotrygon montana*	Mexico	09.29.98.01	16S rDNA	JQ063436
					FusA	JQ063406
23	*C. macrourae* 1	*Leptotila plumbeiceps*	Mexico	10.19.98.04	16S rDNA	JQ063448
24	*C. macrourae* 1	*Leptotila verreauxi*	Mexico	10.19.98.02	16S rDNA	JQ063446
25	*C. macrourae* 2	*Zenaida asiatica*	USA	09.29.98.5	16S rDNA	JQ063447
26	*C. macrourae* 3	*Zenaida macroura*	USA	02.01.99.09	16S rDNA	JQ063450
					GroEL	JQ063393
27	*C. macrourae* 4	*Zenaida galapagoensis*	Galapagos	07.01.99.02	16S rDNA	JQ063425
28	*C. malenkeae*	*Ducula pacifica*	Vanuatu	01.27.04.02	16S rDNA	JQ063417

29	*C. masoni*	*Petrophassa albipennis*	Australia	05.14.03.13	16S rDNA	JQ063441
30	*C. masoni*	*Petrophassa rufipennis*	Australia	01.27.04.12	16S rDNA	JQ063442
					FusA	JQ063403
31	*C. mckeani*	*Ocyphaps lophotes*	Australia	01.20.03.10	16S rDNA	JQ063420
					FusA	JQ063400
					GroEL	JQ063391
32	*C. mjoebergi*	*Geopelia placida*	Australia	05.14.03.17	16S rDNA	JQ063445
					FusA	JQ063404
33	*C. mjoebergi*	*Geopelia striata*	Hawaii	01.20.03.13	16S rDNA	JQ063419
34	*C. mjoebergi*	*Geopelia striata*	Hawaii	03.21.00.05	16S rDNA	
35	*C. passerinae* 2	*Claravis pretiosa*	Mexico	09.29.98.03	16S rDNA	JQ063428
36	*C. passerinae* 2	*Claravis pretiosa*	Mexico	02.01.99.06	16S rDNA	JQ063429
37	*C. paradoxus*	*Lopholaimus antarcticus*	Australia	01.27.04.05	16S rDNA	JQ063423
38	*C. rodmani*	*Geopelia humeralis*	Australia	05.14.03.12	16S rDNA	JQ063443
39	*C. timmermanni*	*Leptotila rufaxilla*	Guyana	01.08.03.07	16S rDNA	JQ063422
40	*C. timmermanni*	*Leptotila rufaxilla*	Guyana	04.24.99.02	16S rDNA	JQ063421
					FusA	JQ063401
41	*C. tschulyschman*	*Columba livia*	USA	05.07.09.01	16S rDNA	JQ063415
42	*C. veigasimoni*	*Phapitreron leucotis*	Phillipines	05.26.99.03	16S rDNA	JQ063438
43	*C. waggermani*	*Patagioenas leucocephala*	USA	11.15.99.08	16S rDNA	JQ063409
44	*C. waiteae*	*Columba leucomela*	Australia	01.27.04.08	FusA	JQ063399
					GroEL	JQ063389
45	*C. waltheri*	*Geotrygon frenata*	Peru	01.20.03.04	16S rDNA	JQ063418

46	*C. wombeyi*	*Geophaps plumifera*	Australia	01.08.03.16	16S rDNA	JQ063410
47	*Physconelloides zenaidurae*	*Zenaida macroura*	USA	N/A	16S rDNA	JQ063413
