# Supplementary material for: Phylogenetic analysis of symbionts in feather-feeding lice of the genus Columbicola: evidence for repeated symbiont replacements
Source: BMC Evol Biol. 2013 May 31;13:109. doi: 10.1186/1471-2148-13-109 (PMC3724504; doi:10.1186/1471-2148-13-109)
Supplement: Additional file 6 — Additional DNA sequences used in the current study. Genbank accession numbers of additional sequences used in phylogenetic analyses in the current study. [file 1471-2148-13-109-S6.pdf]

Isolate	16S rDNA accession #	FusA accession #	GroEL accession#
Buchnera aphidicola strain APS	BA000003	BA000003	BA000003
"*Candidatus* Arsenophonus arthropodicus"	DQ115536		
"*Candidatus* Blochmannia floridanus"	BX248583.1	BX248583.1	BX248583.1
"*Candidatus* Blochmannia pennsylvanicus" strain BPEN	CP00016	CP00016	CP00016
Endosymbiont of *Haematopinus eurysternus*	DQ076661		
Endosymbiont of *Pediculus capitis*	DQ076659		
Endosymbiont of *Pediculus humanus*	DQ076660		
Endosymbiont of *Polyplax serrata*	DQ076667		
Enterobacter endosymbiont of *Metaseiulus occidentalis* clone pAJ240	AY753173		
Enterobacter hormaechei subsp. *steigerwaltii* strain EN562T	AJ853890		
Dickeya dadantii strain S3-1	AY360397		
Escherichia coli K12	U00096	U00096	U00096
Pantoea agglomerans strain NCTC9381T	AJ251466		
Photorhabdus luminescens subsp. *laumondii* TTO1.	BX571859		
Primary endosymbiont of *Sitophilus granarius*	AY126638		
Primary endosymbiont of *Sitophilus oryzae*	AF548142	JX524200	AF005236.1
Primary endosymbiont of *Sitophilus rugicollis*	AY126639		
Primary endosymbiont of *Sitophilus zeamais*	AF548137		
Primary symbiont of *Pseudolynchia canariensis*	DQ115535		
Salmonella enterica subsp. *enterica* serovar *typhimurium*	AP011957	AP011957	AP011957.1
Secondary endosymbiont of *Bactericera cockerelli*	AF263557		
Secondary endosymbiont of *Cantao ocellatus*	AB541010		

Secondary endosymbiont of *Craterina melbae* CMS06	EF174495		
Secondary endosymbiont of *Curculio sikkimensis*, isolate S86_4	AB541010		
Secondary endosymbiont of *Paracoccus nothofagicola*	AF476109		
Shigella flexneri 2002017	CP001383	CP001383	CP001383
Sodalis glossinidius endosymbiont of tsetse *Glossina morsitans*.	AF548135	AP008232	AF326971.1
Strain HS	JX444565	JX524201	JX444566
Vibrio cholerae MJ-1236	CP001486.1	CP001486.1	CP001486.1
Wigglesworthia glossinidia endosymbiont of *Glossina brevipalpis*	BA00021	BA00021	BA00021
Yersinia pestis strain KIM	AE009952	AE009952	AE009952.1
